# Supplementary material for: Association between COVID-19 vaccination, SARS-CoV-2 variants, and post COVID-19 condition: A cross-sectional study
Source: PLoS One. 2025 Dec 2;20(12):e0336929. doi: 10.1371/journal.pone.0336929 (PMC12671788; doi:10.1371/journal.pone.0336929)
Supplement: S1 Table — Abbreviations: UCSD, University of San Diego California. GAD-2, Generalized Anxiety Disorder-2; PHQ-2, Patient Health Questionnaire-2. ◊ The full range of scores for the PROMs are as follows, with higher scores reflecting more symptoms: FSS (sum or mean) (0–63 or 0–7), UCSD (0–120), PTSD (0–5), GAD-2 (0–6), PHQ-2 (0–6). To aid in the interpretation of PROM values, the following normal thresholds have been identified in healthy populations: FSS ≤ 3 (11), PTSD < 4 (14 [33]), GAD-2 < 3 (12 34 ), PHQ-2 < 3 (13 [34]). * Baseline UCSD scores could not be reported due to missing data. For questionnaires with missing data, UCSD scores were considered abnormal if the values from the completed questions were ≥10/120 (a validated threshold representing the presence of significant dyspnea). (DOCX) [file pone.0336929.s001.docx]

| **Supplemental Table 1. Baseline PROM questionnaire responses (n = 1587).^◊^** | | | | | |
| --- | --- | --- | --- | --- | --- |
| Characteristic | Total  (n = 1587) | Vaccination Status | | | |
|  |  | Unvaccinated  (n = 739) | Partially vaccinated  (n = 126) | Fully vaccinated  (n = 178) | Vaccinated after  (n = 544) |
| **Fatigue Severity Score (FSS)** |  |  |  |  |  |
| Proportion with abnormal FSS score, n (%) | 1093 (69) | 483 (65) | 102 (81) | 150 (84) | 358 (66) |
| Baseline FSS score, median (Q1-Q3) | 48 (33-59) | 46 (31-57) | 56 (44-62) | 56 (45-62) | 45 (29-57) |
| **UCSD Dyspnea Score** |  |  |  |  |  |
| Proportion with abnormal UCSD score, n (%)* | 1236 (78) | 562 (76) | 109 (87) | 151 (85) | 414 (76) |
| **Post-traumatic Stress Disorder Score (PTSD)** |  |  |  |  |  |
| Proportion with abnormal PTSD score, n (%) | 333 (21) | 141 (19) | 30 (24) | 38 (21) | 124 (23) |
| Baseline PTSD score, median (Q1-Q3) | 0 (0-2) | 0 (0-2) | 1 (0-3) | 0 (0-2) | 0 (0-2) |
| **Anxiety Screen Score (GAD-2)** |  |  |  |  |  |
| Proportion with abnormal anxiety screen, n (%) | 520 (33) | 229 (31) | 52 (41) | 72 (40) | 167 (31) |
| Baseline anxiety screen, median (Q1-Q3) | 2 (0-3) | 2 (0-3) | 2 (1-4) | 2 (0-4) | 2 (0-3) |
| **Depression Screen Score (PHQ-2)** |  |  |  |  |  |
| Proportion with abnormal depression screen, n (%) | 484 (30) | 205 (28) | 48 (38) | 70 (39) | 161 (30) |
| Baseline depression screen score, median (Q1-Q3) | 2 (0-3) | 2 (0-3) | 2 (1-4) | 2 (1-4) | 2 (0-3) |
| Abbreviations: UCSD, University of San Diego California; GAD-2, Generalized Anxiety Disorder-2; PHQ-2, Patient Health Questionnaire-2.  ^◊^ The full range of scores for the PROMs are as follows, with higher scores reflecting more symptoms: FSS (sum or mean) (0-63 or 0-7), UCSD (0-120), PTSD (0-5), GAD-2 (0-6), PHQ-2 (0-6). To aid in the interpretation of PROM values, the following normal thresholds have been identified in healthy populations: FSS ≤ 3 (11), PTSD < 4 (14,33), GAD-2 < 3 (12,34), PHQ-2 < 3 (13,34).  * Baseline UCSD scores could not be reported due to missing data. For questionnaires with missing data, UCSD scores were considered abnormal if the values from the completed questions were ≥10/120 (a validated threshold representing the presence of significant dyspnea). | | | | |  |
