# Supplementary material for: Association between COVID-19 vaccination, SARS-CoV-2 variants, and post COVID-19 condition: A cross-sectional study
Source: PLoS One. 2025 Dec 2;20(12):e0336929. doi: 10.1371/journal.pone.0336929 (PMC12671788; doi:10.1371/journal.pone.0336929)
Supplement: S2 Table — Participants missing variant data (n = 453), Wild type (n = 361). (DOCX) [file pone.0336929.s002.docx]

| **Supplemental Table 2. SARS-CoV-2 Lineage and Variant Data (n = 773)** | |
| --- | --- |
| **Variant (Lineage)** | **Frequency (%)** |
| **Alpha**  (B.1.1.7) | 198 (26) |
| **Delta**  (AY.103, AY.113, AY.126, AY.18, AY.25.1, AY.25.3, AY.26, AY.27, AY.35, AY.4, AY.4.3, AY.44, AY.65, AY.74, AY.93, B.1.617.2) | 184 (24) |
| **Gamma**  (P.1, P.1.14, P1/B.1.351) | 250 (32) |
| **Omicron**  (B.1.1.529, BA.1, BA.1.1, BA.1.1.16, BA.1.1.6, BA.1.15, BA.1.17.2, BA.1.120, BA.2, BA.2.12.1, BA.2.3, BA.5.5) | 51 (7) |
| **Other non-VOC strains**  Eta (B.1.525)  Epsilon (B.1.429)  Kappa (B1.617.1)  Zeta (P.2)  Lineage A (A.23.1) | 90 (11) |
| Participants missing variant data (n = 453)  Wild type (n = 361) | |
