# Supplementary material for: Association between COVID-19 vaccination, SARS-CoV-2 variants, and post COVID-19 condition: A cross-sectional study
Source: PLoS One. 2025 Dec 2;20(12):e0336929. doi: 10.1371/journal.pone.0336929 (PMC12671788; doi:10.1371/journal.pone.0336929)
Supplement: Attachment — Submitted filename: Response to Reviewers.docx [file pone.0336929.s004.docx]

August 21^st^, 2025

We thank the Editors and Reviewers for their review and thoughtful comments. We hope we have addressed this feedback to their satisfaction.

Sincerely,

Saif El-Akkad, on behalf of the authors

**Editor’s Comments:**

1. Please ensure that your manuscript meets PLOS ONE's style requirements, including those for file naming.

Response: Thank you for sharing the need for additional style and formatting requirements. Using the provided templates the following changes were made:

- Title page heading changed to size 18 and bolded.
- All author affiliations modified to include department, division, institution, city, province and country.
- Only the email for the corresponding author is now provided.
- Additional text from the title page (word count, figure/table count, keywords) has been removed from title page.
- Abstract has been shortened to 300 words as outlined in submission guidelines.
- Changed headings and subheadings to requested format for level 1 and level 2 headings.
- Changed “figure” to “fig” as requested throughout the manuscript.
- Table 3 and the Figure 3 heading now appear after the paragraph in which they are mentioned.
- Table S1 is now referenced in the manuscript body (page 8): “Baseline PROM scores were also collected and the proportion of participants who reported abnormal scores was calculated (Table S1).”
- An acknowledgements heading is now provided prior to the references, other headings (list of abbreviations, ethics statement, availability of data and materials, conflicts of interest, funding statements and author contributions) have been moved to their respective sections in the manuscript body or removed if not required by PLOS ONE’s formatting requirements.
- Supporting information section now includes both the S1 and S2 tables titles and legends in the requested format.
- Figures and supplemental table files have been renamed according to requested format.

2. We note that you have indicated that there are restrictions to data sharing for this study. For studies involving human research participant data or other sensitive data, we encourage authors to share de-identified or anonymized data. However, when data cannot be publicly shared for ethical reasons, we allow authors to make their data sets available upon request. For information on unacceptable data access restrictions, please see http://journals.plos.org/plosone/s/data-availability#loc-unacceptable-data-access-restrictions.

Before we proceed with your manuscript, please address the following prompts:

a) If there are ethical or legal restrictions on sharing a de-identified data set, please explain them in detail (e.g., data contain potentially identifying or sensitive patient information, data are owned by a third-party organization, etc.) and who has imposed them (e.g., a Research Ethics Committee or Institutional Review Board, etc.). Please also provide contact information for a data access committee, ethics committee, or other institutional body to which data requests may be sent.

b) If there are no restrictions, please upload the minimal anonymized data set necessary to replicate your study findings to a stable, public repository and provide us with the relevant URLs, DOIs, or accession numbers. Please see http://www.bmj.com/content/340/bmj.c181.long for guidelines on how to de-identify and prepare clinical data for publication. For a list of recommended repositories, please see https://journals.plos.org/plosone/s/recommended-repositories. You also have the option of uploading the data as Supporting Information files, but we would recommend depositing data directly to a data repository if possible.

Please update your Data Availability statement in the submission form accordingly.

Response: According to the University of British Columbia Clinical Research Ethics Board (UBC CREB), we are prohibited from sharing data publicly as there is potentially sensitive information. Data requests may be sent to the UBC CREB at the following address: Room 210, Research Pavilion - 828 West 10th Ave, Vancouver, BC Canada V5Z1M9

The Data Availability statement in the submission form has been updated to the following: “Data requests may be sent to the UBC CREB at the following address: Room 210, Research Pavilion - 828 West 10th Ave, Vancouver, BC Canada V5Z1M9”

3. You have indicated that data is available from [svitlana.franchuk@ors.ubc.ca].  Please can we ask you to provide us with a general contact email address for the data requests, so readers can request access in perpetuity. If a general email is not available, please provide a link to a website where readers can obtain access to data.

Response: Data requests may be sent to the UBC CREB at the following address: Room 210, Research Pavilion - 828 West 10th Ave, Vancouver, BC Canada V5Z1M9. A general email is not available, but up-to-date contact information can be found on the UBC CREB website: <https://researchethics.ubc.ca/clinical-research-ethics/contact-creb>

4. Thank you for stating the following in your manuscript:

“AWW received funding from the Canadian Lung Association (CLA) / Canadian Institutes of Health Research (CIHR) Respiratory Health Effects of PCC Grant (Funding reference 181075). The funders had no role in study design, data collection and analysis, decision to publish, or preparation of the manuscript. “

We note that you have provided additional information within the Acknowledgements Section that is not currently declared in your Funding Statement. Please note that funding information should not appear in the Acknowledgments section or other areas of your manuscript. We will only publish funding information present in the Funding Statement section of the online submission form.

Please remove any funding-related text from the manuscript and let us know how you would like to update your Funding Statement. Currently, your Funding Statement reads as follows:

“AWW received funding from the Canadian Lung Association (CLA) / Canadian Institutes of Health Research (CIHR) Respiratory Health Effects of PCC Grant (Funding reference 181075). The funders had no role in study design, data collection and analysis, decision to publish, or preparation of the manuscript.”

Please include your amended statements within your cover letter; we will change the online submission form on your behalf.

Response: The funding statement that was previously provided in the acknowledgements section has been removed. The current funding statement is correct and there are no amendments.

5. Your ethics statement should only appear in the Methods section of your manuscript. If your ethics statement is written in any section besides the Methods, please delete it from any other section.

Response: The ethics statement has been removed from the acknowledgement section of the manuscript and is now only mentioned in the methods section as requested.

**Reviewer: 1**

No additional comments to address.

**Reviewer: 2**

R2C1. While the authors adjust for several baseline characteristics—including age, sex, and comorbidities—the inclusion of time-dependent covariates would strengthen the analysis. Specifically, stratifying by time since infection or time since last vaccination dose may provide a more nuanced understanding of how temporal dynamics influence post-COVID outcomes.

R2R1: Thank you for this feedback. The median time from infection to completion of PROM questionnaires was approximately 100 days (with interquartile range of 50 days) and similar between the vaccination groups. Time-varying covariates were not included given this relatively short time interval. We have included the median times between infection and questionnaire completion in Table 1.

R2C2. Although the multivariable models account for several relevant factors, residual confounding remains a concern, particularly with respect to COVID-19 illness severity. Disease severity is likely to influence both the risk of developing post-COVID conditions (PCC) and the probability of being referred to a post-COVID recovery clinic.

R2R2: Patients were stratified into those who were hospitalized and were not hospitalized to address disease severity. We have added the following sentence discussing the impact of disease severity as a potential confounder and how we attempted to minimize its impact on the study (discussion paragraph 5).

“We also stratified patients based on whether or not they were hospitalized for COVID-19 as the severity of acute illness may increase the development and severity of PCC (2).”

R2C3. The absence of a non-infected comparison group precludes estimating the absolute risk or excess burden of post-COVID conditions. Without a reference population of individuals who were never infected, it is impossible to determine whether the prevalence of abnormal PROMs (e.g., fatigue, dyspnea, anxiety) is elevated beyond what might be expected in the general population or among those with similar comorbidities but without SARS-CoV-2 exposure.

R2R3: Thank you for raising this valid concern, we agree that this is a key limitation of the study and have added the text below discussing this limitation and how we attempted to minimize its impact on the study (discussion paragraph 5).

“This study is limited by the absence of PROM data prior to COVID-19 infection and lack of a control cohort, which restricts our ability to attribute the presence of abnormal PROMs to PCC. We attempted to focus in on the relationship between COVID-19 infection and abnormal PROMs by adjusting for comorbidities to address the impact that pre-existing conditions and impaired health may have on PROMs.”

We have also added additional text that aids in the interpretation of the reported PROM values and their comparison to normative PROM values reported in the general population (Supplemental Table 1).

“The full range of scores for the PROMs are as follows, with higher scores reflecting more symptoms: FSS (sum or mean) (0-63 or 0-7), UCSD (0-120), PTSD (0-5), GAD-2 (0-6), PHQ-2 (0-6). To aid in the interpretation of PROM values, the following normal thresholds have been identified in healthy populations: FSS ≤ 3 (11), PTSD < 4 (14,33), GAD-2 < 3 (12,34), PHQ-2 < 3 (13,34).”

R2C4. Considering that this is a clinic-based cohort of COVID-19 survivors, there are additional selection biases (referral and survival bias) and generalizability concerns that should be clearly acknowledged in the paper.

R2R4: We thank reviewer 2 for raising this suggestion. Concerns around referral bias and the generalizability of this cohort have been addressed in the text below (discussion paragraph 5).

“Additionally, while the PCRC cohort represents a large cohort of approximately 3800 patients with COVID-19 who have completed validated questionnaires and physiological testing, there is potential referral bias (e.g., care providers in rural locations may not be aware of the PCRCs and those with mild disease may not be referred to the PCRC). While this population may not be fully generalizable, it does allow us to study patients with sustained and severe PCC who would most likely require and benefit from further investigations and treatment.”
