# Supplementary material for: Association between COVID-19 vaccination, SARS-CoV-2 variants, and post COVID-19 condition: A cross-sectional study
Source: PLoS One. 2025 Dec 2;20(12):e0336929. doi: 10.1371/journal.pone.0336929 (PMC12671788; doi:10.1371/journal.pone.0336929)
Supplement: Attachment — Submitted filename: Reviewer Responses Round 2.docx [file pone.0336929.s005.docx]

October 11^th^, 2025

We thank the Editors and Reviewers for their review and thoughtful comments. We hope we have addressed this feedback to their satisfaction.

Sincerely,

Dr. Saif El-Akkad, on behalf of the authors

**Editor’s Comments:**

1. The following additional editor comments were made:
   a. COVID infection -> SARS-CoV-2 infection
   b. COVID-19 infection -> SARS-CoV-2 infection
   c. COVID vaccination -> COVID-19 vaccination
   d. Demonstrated -> found

Response: Thank you for sharing the need for additional style and formatting requirements. The following changes were made:

- COVID infection has been changed to SARS-CoV-2 infection.
- COVID-19 infection has been changed to SARS-CoV-2 infection.
- COVID vaccination has been changed to COVID-19 vaccination.
- Demonstrated has been changed to found.

2. Please describe definition of variant of concern (VOC) duration.

Response: Text has been added to clarify the time periods during which variants of concern circulated and the period during which they were studied (methods paragraph 4):

VOCs circulated in British Columbia during the following approximate time periods: alpha (January – June 2021), gamma (February – July 2021), delta (May 2021 – December 2021) and omicron (December 2021 onwards) (17). Data were limited to these four dominant VOCs based on data availability and categorization used by the BCCDC Public Health Laboratory (PHL) during the study duration (March 2020 to October 2022).

Please let us know if we did not satisfactorily describe the VOC duration with our response above. We would be happy to re-address this comment with further clarification on what VOC duration refers to.

**Reviewer Comments:**

No additional comments to address.
